# Effects of *Annurca* Flesh Apple Polyphenols in Human Thyroid Cancer Cell Lines

**DOI:** 10.1155/2022/6268755

**Published:** 2022-02-17

**Authors:** Francesca Maria Orlandella, Peppino Mirabelli, Anna Elisa De Stefano, Paola Lucia Chiara Iervolino, Neila Luciano, Stefania D'Angelo, Giuliana Salvatore

**Affiliations:** ^1^IRCCS SYNLAB SDN, Naples, Italy; ^2^Dipartimento di Scienze Motorie e del Benessere, Università di Napoli Parthenope, Naples, Italy; ^3^CEINGE-Biotecnologie Avanzate S.c.a.r.l., Naples, Italy; ^4^Dipartimento di Scienze Biomediche Avanzate, Università di Napoli Federico II, Naples, Italy

## Abstract

Among natural macromolecules, the polyphenol extract from *Annurca* flesh (*A*FPE) apple could play a potential therapeutic role for a large spectrum of human cancer also by exerting antioxidant properties. Thyroid cancer is a common neoplasia in women, and it is in general responsive to treatments although patients may relapse and metastasize or therapy-related side effects could occur. In this study, we explored the effects of *A*FPE on papillary (TPC-1) and anaplastic (CAL62) thyroid cancer cell line proliferation and viability. We found that *A*FPE exposure induced a reduction of cell proliferation and cell viability in dose-dependent manner. The effect was associated with the reduction of phosphorylation of Rb protein. To study the mechanisms underlying the biological effects of *A*FPE treatment in thyroid cancer cells, we investigated the modulation of miRNA (miR) expression. We found that *A*FPE treatment increased the expression of the miR-141, miR-145, miR-200a-5p, miR-425, and miR-551b-5p. Additionally, since natural polyphenols could exert their beneficial effects through the antioxidant properties, we investigated this aspect, and we found that *A*FPE treatment reduced the production of reactive oxygen species (ROS) in CAL62 cells. Moreover, *A*FPE pretreatment protects against hydrogen peroxide-induced oxidative stress in thyroid cancer cell lines. Taken together, our findings suggest that *A*FPE, by acting at micromolar concentration in thyroid cancer cell lines, may be considered a promising adjuvant natural agent for thyroid cancer treatment approach.

## 1. Introduction

Thyroid cancer, the most common endocrine tumor, is traditionally classified into different subtypes: papillary, follicular, Hürthle cells, medullary, and undifferentiated or anaplastic carcinoma [1]. The most frequent form is papillary thyroid carcinoma (PTC), for which the standard treatments are often successful, although a fraction of patients could develop drug resistance, recurrence, and metastasis [2, 3]. The most aggressive subtype is undifferentiated or anaplastic thyroid cancer (ATC) characterized by poor overall survival [4, 5]. Therefore, it is essential to find alternative therapeutic strategies to treat unresponsive form.

Among plant-derived compounds, phytochemicals exert multiple physiological functions and have been shown to be helpful in the protection against DNA damage and genomic instability [6, 7]. Several phytochemicals (as resveratrol or taxol analogues) isolated from medicinal plants are also commonly used for therapy against various types of cancer [8–10]; additionally, other phytochemicals (as curcumin) can decrease side effects of chemotherapy and improve quality of life in cancer patients [6, 11].

Polyphenols, the most abundant natural macromolecules among phytochemicals, are chemical substances enclosed within the plants that show several anticancer properties through the inhibition of cell proliferation and motility and the induction of apoptosis; in addition, they exert antioxidant and antiangiogenesis activity with few side effects [12–20]. Polyphenols are also able to regulate the immunity system by reducing the expression of cytokines, by inactivating nuclear factor kappa-light-chain-enhancer of activated B cells (NF-*κ*B) and by inhibiting the mitogen-activated protein kinase (MAPK) and phosphatidylinositide 3-kinases/protein kinase B (PI3K/AKT), mammalian target of rapamycin complex 1 (mTORC1), and Janus chinasi (JAK)/signal transducer and activator of transcription (STAT) [13]. Finally, evidences show that polyphenol compounds are able to modulate the epithelial-to-mesenchymal transition (EMT) by upregulating the expression of epithelial markers such as E-cadherin and by inhibiting the expression of mesenchymal markers [21, 22]. Based on these data, polyphenol consumption represents one of the strategies proposed by clinical chemoprevention in the context of the predictive, preventive, personalized medicine (3PM) [7].

In this frame, *in vitro* and *in vivo* studies have been performed in different types of thyroid cancer, and several reviews well summarize the beneficial effects that the various phytochemicals exert in thyroid cancer cells [14, 23–28].

Apple fruits contain high levels of polyphenols and other phytochemical compounds. The biological effects resulting by the consumption of apple polyphenols have been investigated in numerous studies showing its antioxidant and antiangiogenic ability [29]. Apple fruit displays also cardioprotective effects, antiarteriosclerosis, and antihypertensive activity by reducing low-density lipoprotein oxidation and also by decreasing glucose levels and lipid uptake [30–32]. Furthermore, among the benefits of apple fruit, there is also the capability to contribute to prevent tumor formation in different types of human cancer [29, 33–35].

*Malus pumila* Miller cv. *Annurca* is an apple variety with a “Protected Geographical Indication” of the Campania region [36] accounting for approximately 5% of Italian apple production. Compared to other varieties, it is richer in catechin, epicatechin, and chlorogenic acid endowing a stronger antioxidant activity [37]. We have previously shown the antiproliferative effect of flesh polyphenol extract from *Annurca* flesh apple in human HaCaT keratinocytes [38] and in human breast carcinoma cells [39–43].

Within this frame, in this paper, we have investigated the role of polyphenol extract activities from *Annurca* flesh polyphenolic extract (*A*FPE) on the malignant phenotype of human thyroid cancer cells.

## 2. Materials and Methods

### 2.1. Apple Samples

*Annurca* apple (*Malus pumila* cv. *Annurca)* fruits (weight ~100 g) were collected in Giugliano (Naples, Italy) in October, immediately after the fruit (green peel) had been harvested. Fruits were reddened in the “*melai*” according to peculiar proceedings and then used [35, 36].

### 2.2. *A*FPE Polyphenol Extraction and Characterization of Polyphenol Content

*A*FPE extraction from *Annurca* apple was carried out as previously indicated by D'Angelo et al. [44]. Briefly, 40 grams of *Annurca* apple flesh were homogenized, using a Tefal Rondo 500 homogenizer, in 40 ml solution of 80% methanol and 20% water plus 180 mM HCl, for 10 minutes. After centrifugation (18.000 x g for 30 minutes), the slurry was dried under vacuum by Univapor Concentrator Centrifuge (model Univapo 100 H-Uni Equip, Munich, Germany). The dried sample was mixed in 10 ml of phosphate-buffered saline (PBS) and frozen at -80°C [44, 45].

The total polyphenolic content in the extract obtained was assessed by the Folin-Ciocalteu colorimetric method [43]. Briefly, 100 *μ*l of the extracts were mixed with the Folin–Ciocalteu phenol reagent (500 *μ*l), deionized water (900 *μ*l), and Na_2_CO_3_ (7.5% *w*/*v*, 4 ml). The sample was incubated 1 hour at room temperature. Then, the absorbance at 765 nm was measured using a Cary ultraviolet–visible spectrophotometer (Varian) (Agilent Technologies, Santa Clara, USA) and compared to a standard curve of catechin solutions [43, 44]. To provide *A*FPE an arbitrary molar concentration, since it is a mixture of several phenolic compounds with different molecular weights, its polyphenol concentration was expressed as milligrams of catechin equivalents (EqC)/100 g of *Annurca* flesh fresh weight.

The chemical characterization of *A*FPE was performed as reported by Vuoso et al. [43, 45]. Separation of polyphenols was done by HPLC using reversed-phase chromatography on a 5 *μ*m column Kromasil C18 column (150 × 4.6 mm), using a Beckman Apparatus (Gold-126) with a UV detector fixed at 278 nm. On the basis of the retention time of standard references, the main *o*-diphenols (+)-catechin, (-)-epicatechin, and chlorogenic acid were identified [43].

### 2.3. Cell Cultures

The human anaplastic (CAL62) and papillary (TPC-1) thyroid cancer cell lines were grown in Dulbecco's modified Eagle's medium (DMEM) (Thermo Fisher Scientific, Waltham, MA, USA) containing 10% fetal bovine serum (FBS), 100 U/ml penicillin, 100 mg/ml of streptomycin, and 200 mM of L-glutamine (Thermo Fisher Scientific) at 37°C in a humidified incubator containing 5% CO_2_ and 95% humidity. All experiments were performed in medium containing 10% FBS.

### 2.4. Cell Counting

Cell counts were evaluated by direct cell counting in trypan blue reagent. In detail, TPC-1 and CAL62 cells were plated in 6-well plates at a density of 5 × 10^4^ and 3 × 10^4^ cells, respectively, and kept in DMEM supplemented with 10% of FBS. The day after plating, the medium was replaced by fresh complete medium with different concentrations (50, 100, 250, 500, 750, and 1000 *μ*M EqC, i.e., 14.5, 29, 70, 145, 210, and 290 *μ*g EqC/ml) of *A*FPE while the untreated cells were used as a control.

To determine the number of live and dead cells, 24 hours after treatments, CAL62 and TPC-1 cells were collected by trypsinization, resuspended in PBS, and stained with 0.4% trypan-blue (Bio-Rad, Richmond, VA, USA) according to manufacturer's instructions and counted with a TC10™ Automated Cell Counter (Bio-Rad). Then, the percentage of viability (viability %) was obtained dividing the number of viable cells by the number of total cells (viable + dead cells).

### 2.5. Cell Cycle Analysis

Cell cycle progression was determined by flow cytometry using the Cytoflex instrument purchased from Beckman Coulter (Milan, Italy). To this aim, cells were stained using Coulter DNA PREP Reagents Kit, (Beckman Coulter) according to manufacturer's instructions. Briefly, TPC-1 and CAL62 (5 × 10^4^ cells) were treated with *A*FPE for 24 hours at the doses of 250 and 500 *μ*M at 37°C with 5% CO2. After cellular detachment, cells were incubated with DNA PREP LPR reagent for 15 minutes and then with DNA PREP STAIN, containing 50 *μ*g/ml of propidium iodide, for 1 hour. TPC-1 and CAL62 stained cells were acquired using the Cytoflex flow cytometer (Beckman Coulter). Cell cycle progression was analyzed using Kaluza Analysis Software 2.1 (Beckman Coulter) applying the Michael Fox algorithm.

### 2.6. Western Blot

Proteins were lysed in ice using JS Buffer supplemented with protease inhibitors and quantified using a Bradford assay (Bio-Rad) according to standard protocols.

Protein lysates were separated in a sodium dodecyl sulphate–polyacrylamide gel electrophoresis (SDS-PAGE); then, the nitrocellulose membranes were hybridized with the following antibodies: rabbit polyclonal phospho-Rb (Ser795) diluted 1 : 1000 (#9301, Cell Signaling, Danvers, USA) and mouse monoclonal anti-*α*-Tubulin diluted 1 : 10000 (T-9026, Sigma-Aldrich, St. Louis, USA). Secondary anti-mouse and anti-rabbit antibodies coupled to horseradish peroxidase were diluted 1 : 3000 and purchased from Bio-Rad. The bands were detected by an enhanced chemiluminescence detection kit (ECL, Thermo Fisher Scientific) and analyzed by the Image Lab™ Software (Bio-Rad).

### 2.7. RNA Extraction and q-RT-PCR

Total RNA containing miRNAs was extracted from CAL62 cells untreated or treated with *A*FPE 500 *μ*M EqC for 24 hours using TRIzol reagent (Thermo Fisher Scientific) in accordance with standard procedures and quantified using a NanoDrop spectrophotometer (Thermo Fisher Scientific).

cDNA was reverted using a miScript II RT Kit (Cat. Number 218161) following manufacturer's instructions.

The mature miRNA expression was determined by the real-time PCR (q-RT-PCR) using SYBR Green PCR Master Mix (Cat. Number 218073). The following miRNA-specific miScript primers (Cat. Number 218300) were used: miR-141 (ID MS00003507), miR-145 (ID MS00003528), miR-200a-5p (ID MS00009009), miR-551b-5p (ID MS00010157), and miR-425 (ID MS00009695). All reagents were purchased from Qiagen (Hilden, Germany).

The Ct-value of miRNAs was normalized with snU6 (ID MS00029204) used as endogenous control, and the fold changes were calculated using the formula 2^−ΔΔCt^. Three independent experiments were performed in triplicate.

### 2.8. Measurements of ROS Production

To quantitatively assess the reactive oxygen species (ROS) production, the DCFDA/H2DCFDA- Cellular ROS Assay Kit (ab113851) purchased from Abcam (Cambridge, UK) was used. Briefly, CAL62 cells were seeded in 96-well plate at the density of 5 × 10^3^ cells/well in FBS-supplemented medium and allowed to attach overnight. Then, cells were incubated with *A*FPE (250 or 500 *μ*M EqC) for 24 hours or with H_2_O_2_ (0.05, 0.5, or 1 mM) for 4 hours, alone or in combination. Cells were then washed once with PBS, labeled with the fluorogenic dye 2′,7′–dichlorofluorescin diacetate (DCFDA) for 45 minutes protected from light, washed with Buffer 1X (provided in the kit), and analyzed on a microplate reader (EnSpire Multimode plate reader by PerkinElmer, Milan, Italy) with the excitation at 485 nm and the emission at 535 nm according to manufacturer's instructions. ROS production was measured every 30 minutes for 4 hours. The unlabeled CAL62 cells were analyzed and used as negative controls.

### 2.9. Cell Death Analysis

For the discrimination of live/dead cells, we used 7-AAD staining. Specifically, thyroid cancer cells (TPC-1 and CAL62) were pretreated with 500 *μ*M of *A*FPE for 20 hours; then, the peroxide hydrogen (H_2_O_2_) at concentration of 1 mM was added to the medium for other 4 hours. At the end of the treatments, cells were detached by trypsin, collected by centrifugation, and resuspended in 100 *μ*l DPBS supplemented with 1% FBS containing 5 *μ*l of a 10 *μ*g/ml 7-AAD solution for 10 minutes in the dark before analysis. For the discrimination of early, late apoptosis and of necrosis, CAL62 cells untreated or treated were also stained using Annexin V (Beckman Coulter) according to the manufacturer and analyzed.

### 2.10. Statistical Analysis

The statistical analysis was performed with the GraphPad Prism 9 software (La Jolla, CA, USA), and the data are shown as the mean ± standard error of the mean (SEM). Differences were regarded as significant when *p* < 0.05.

For the differences between untreated and *A*FPE treatment group, Student's *t* tests were used, while for the multiple comparisons between the four experimental groups (untreated, *A*FPE, H_2_O_2_, and *A*FPE/H_2_O_2_), ANOVA was calculated followed by a post hoc test (Bonferroni).

## 3. Results

### 3.1. Determination of Polyphenolic Content in *A*FPE

The total polyphenol amount in *A*FPE was quantified by Folin-Ciocalteu assay and resulted to be approximately 126 mg of catechin per 100 g of apple flesh; this concentration was comparable to that measured in other studies [40, 42, 44]. Then, we analyzed the polyphenols profile by HPLC in which the main *o*-diphenols (+)-catechin, (-)-epicatechin, and chlorogenic acid were detected on the basis of the retention time of standard references [43]. The data agree with the literature [35, 36, 43, 44].

### 3.2. *A*FPE Inhibits the Viability and Cell Cycle Progression of Anaplastic Thyroid Cancer Cell Line

Anaplastic thyroid cancer cell line, CAL62, was treated with increasing doses of *A*FPE (from 50 to 1000 *μ*M EqC) for 24 hours, and the number of live and dead cells and cell viability were simultaneously evaluated by counting cells after trypan blue staining.

**Figure 1:**
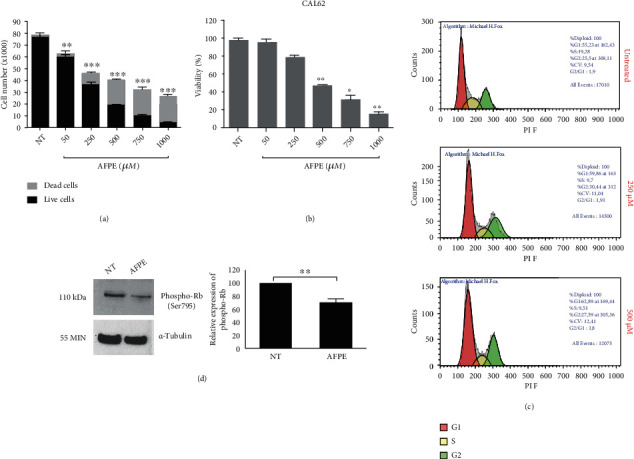
Effects of *A*FPE treatment on proliferation and viability of anaplastic thyroid cancer cell line. (a) Cell counting was performed by trypan blue reagent after treatment with increasing doses of *A*FPE for 24 hours. Black bars represent the viable cells (assessed by trypan blue exclusion), and dashed bars represent the dead cells. (b) The percentage of cell viability (viable cells/total cells) was calculated in CAL62 cell line treated with *A*FPE and counted in trypan blue dye. (c) Cell cycle analysis after propidium iodide (PI) staining of CAL62 untreated (NT) or treated with 250 or 500 *μ*M of *A*FPE EqC for 24 hours. Representative fluorescence-activated cell sorting (FACS) plots are shown applying the Michael Fox algorithm. (d) Protein extracts from CAL62 cells treated with 500 *μ*M of *A*FPE EqC for 24 hours and untreated were analyzed by Western blot using antibodies for phospho-Rb (Ser795) and for *α*-tubulin. Representative figures from at least four independent experiments are shown (left panel). Relative expression level of phospho-protein was calculated using *α*-tubulin as an endogenous control (right panel). Values are expressed as mean of at least three different experiments ± standard error of the mean (SEM). ^∗^*p* < 0.05; ^∗∗^*p* < 0.01; ^∗∗∗^*p* < 0.001 versus control (NT).

As shown in Figure 1(a), the treatment with *A*FPE significantly reduced cell proliferation in a dose-dependent manner; appreciable changes in cell number were observed already at 50 *μ*M of *A*FPE EqC.

As reported in Figure 1(b), viability percentages in CAL62 were approximately 46, 28, and 13% after treatments with 500, 750, and 1000 *μ*M EqC of *A*FPE, respectively, in comparison to untreated (NT) cells that showed approximately 100% of viability.

Additionally, 24 hours after treatments with 250 and 500 *μ*M of *A*FPE, cell cycle progression was analyzed by flow cytometry with propidium iodide staining. Figure 1(c) shows that the dose of 500 *μ*M EqC of *A*FPE induces a slight accumulation of CAL62 cells at G1 phase compared to untreated cells.

### 3.3. *A*FPE Reduced the Phosphorylation of Rb

To better investigate the molecular mechanisms underlying the effects on cell cycle induced by *A*FPE treatments, we analyzed the phosphorylated levels of the cell cycle regulator Rb by Western blot. Consistent with the results obtained by flow cytometry, treatment with 500 *μ*M *A*FPE EqC for 24 hours reduces the phosphorylation of Rb protein in CAL62 cells (Figure 1(d)) compared to control. This result confirms that *A*FPE treatment acts on G1/S phase of the cell cycle through Rb signaling pathway.

### 3.4. Anticancer Effects of *A*FPE are Mediated by Deregulation of miRNA Expression

MicroRNAs (miRNAs) are small molecules able to drive different biological process through downregulation of specific target genes. Deregulation of miRNA expression drives a significant contribution in several human neoplasia, including thyroid cancer development and progression, since they can act as onco-miR or tumor suppressor miRNAs [46, 47]. Notably, in literature, it is also reported that polyphenols exert their anticancer properties by targeting different miRNAs [48, 49]. Thus, it is possible that the anticancer activity exerted by *A*FPE is in part due to the modulation of miRNA expression also in our cell model system.

**Figure 2:**
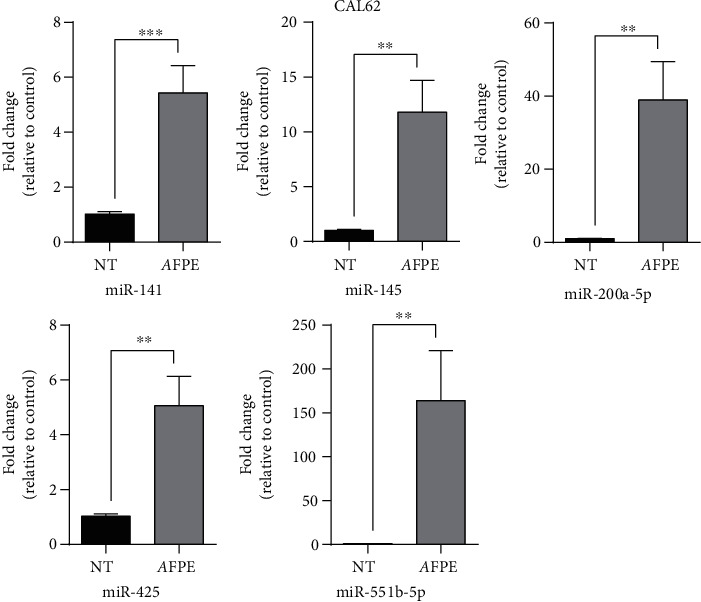
Effects of *A*FPE treatment on miRNA expression levels. The relative expression level of the indicated miRNAs was measured in CAL62 cells untreated (named NT) or treated with 500 *μ*M of *A*FPE EqC for 24 hours by q-RT-PCR. Fold change was calculated using the formula 2^−ΔΔCt^ in which the Ct value of miRNA was normalized with an internal control (snU6). Bar represents the mean ± SEM of three independent experiments performed in triplicate. ^∗∗^*p* < 0.01; ^∗∗∗^*p* < 0.001.

To verify if *A*FPE can modulate miRNA expression levels, CAL62 cells were treated with 500 *μ*M of *A*FPE EqC for 24 hours, and q-RT-PCR was performed to analyze the expression level of several miRNAs that are known to be involved in thyroid carcinogenesis.  Figure 2 shows that CAL62 cells treated with 500 *μ*M of *A*FPE EqC presented an increased expression level of the members of miR-200 family (miR-200a-5p and miR-141) and of miR-145, miR-425, and miR-551-5p.

These data suggest that miRNA targeting is one of the mechanisms modulated by *A*FPE to exert its anticancer activity in thyroid carcinoma cell lines.

### 3.5. *A*FPE Inhibits the Viability and Cell Cycle Progression of Papillary Thyroid Cancer Cell Line

To further confirm the results obtained, we also tested the viability of a papillary thyroid cancer cell line, TPC-1, after treatment with increasing doses of *A*FPE (ranging from 50 to 1000 *μ*M EqC).

The effects obtained on cell proliferation and on cell viability were simultaneously evaluated by counting viable and dead cells with trypan blue reagent after 24 hours from *A*FPE treatments.

**Figure 3:**
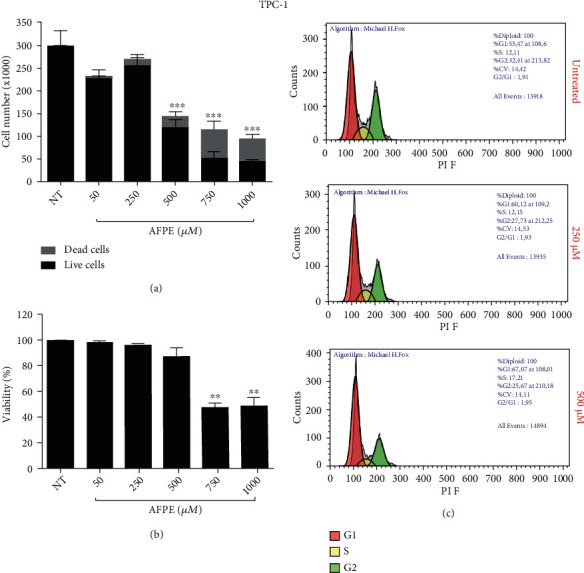
Effects of *A*FPE treatment on the proliferation and viability in papillary thyroid cancer cell line. (a) TPC-1 cells were treated with the indicated doses of *A*FPE, and after 24 hours, cells were collected by trypsinization, stained for 5 minutes with trypan blue, and counted; black bars represent the viable cells, and dashed bars represent the dead cells. (b) After counting cells with trypan blue reagent, the percentage of cell viability (viable cells/total cells) was calculated. Values are expressed as mean of two different experiments ± standard error of the mean (SEM). ^∗∗^*p* < 0.01; ^∗∗∗^*p* < 0.001. (c) Cell cycle analysis after propidium iodide staining was analyzed by flow cytometry in TPC-1 cell line treated with different doses of *A*FPE for 24 hours. Representative fluorescence-activated cell sorting (FACS) plots are shown applying the Michael Fox algorithm.

The results obtained evidence that up to 250 *μ*M *A*FPE EqC, no appreciable changes in cell number and cell viability were observed. The number of live cells was decreased after treatments with higher doses of *A*FPE, and in parallel, the number of dead cells was increased (Figures 3(a) and 3(b)).

Accordingly, as reported in Figure 3(b), when we treated TPC-1 cell line with increasing doses of *A*FPE for 24 hours, the reduction of viability occurred: from approximately 100% in untreated (NT) cells to less than 50% in TPC-1 treated with 750 and 1000 *μ*M EqC of *A*FPE.

The effects of *A*FPE treatments were also evaluated by flow cytometry analysis of the cell cycle distribution. As reported in Figure 3(c), in agreement with the results in ATC cells, *A*FPE induced an accumulation of TPC-1 population in G1 phase compared to untreated cells.

All together, these data unveiled that the proliferation and the viability were reduced in human papillary and anaplastic thyroid cancer cells exposed to *Annurca* apple extracts in a dose-dependent manner.

### 3.6. Antioxidant Effect of *A*FPE on Human Thyroid Cancer Cells

Since the involvement of polyphenol extract from *Annurca* apple fruits in the regulation of reactive oxygen species (ROS) generation is reported [41], we investigated the effects of *A*FPE on oxidative stress.

**Figure 4:**
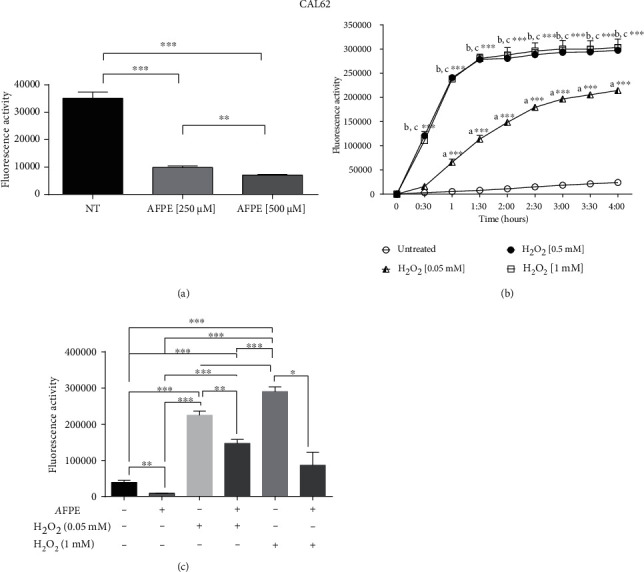
Effects of *A*FPE treatment on the production of reactive oxygen reagents (ROS) in anaplastic thyroid cancer cell line. (a) CAL62 cells untreated or treated for 24 hours with *A*FPE (250 or 500 *μ*M EqC) were stained with the fluorogenic dye DCFDA (2′,7′-dichlorofluorescein diacetate) for ROS detection. Fluorescence activity was read with the excitation at 485 nm and the emission at 535 nm. Significant differences were analyzed using paired *t*-test. (b) ROS quantification within the CAL62 cells treated with different doses of H_2_O_2_ (0.05, 0.5, and 1 mM) was determined using DCFDA. Fluorescence activity was measured every 30 minutes for 4 hours. Significant difference compared to the untreated cells was analyzed using *T*-test. Letter “a” indicates the significant difference between untreated and H_2_O_2_ (0.05 mM), letter “b” significant difference between untreated and H_2_O_2_ (0.5 mM), and letter “c” significant difference between untreated and H_2_O_2_ (1 mM). (c) ROS production in CAL62 cells untreated, treated with *A*FPE (500 *μ*M EqC) for 24 hours, treated with H_2_O_2_ (0.05 or 1 mM) for 4 hours, or pretreated with *A*FPE (500 *μ*M EqC) for 20 hours and then treated with H_2_O_2_ (0.05 or 1 mM) for 4 hours. Statistical analysis was performed using ANOVA followed by a Bonferroni post hoc test. All the data are shown as mean ± SEM of at least three independent experiments in triplicate. ^∗^*p* < 0.05; ^∗∗^*p* < 0.01; ^∗∗∗^*p* < 0.001.

Thus, we initially verified if *A*FPE exerts antioxidant or prooxidant effects in our cell model system. To this aim, CAL62 cell line was treated with 250 and 500 *μ*M of *A*FPE EqC for 24 hours, and the production of reactive oxygen species (ROS) within the live cells was quantitatively assessed by the DCFDA/H2DCFDA-Cellular ROS assay kit. As shown in Figure 4(a), treatment with 250 *μ*M of *A*FPE EqC decreased the production of ROS of ~3.6-fold, while 500 *μ*M of *A*FPE EqC treatment reduces the production of ROS of ~5-fold compared to untreated (NT) cells. These data suggest that *A*FPE exerts antioxidant effects in thyroid cancer cell line in dose-dependent manner.

Next, we verified if *A*FPE treatment is also able to protect thyroid cancer cells from extracellular stress signal such as the exposure to hydrogen peroxidase (H_2_O_2_). To this aim, first, we treated CAL62 cells with different doses of H_2_O_2_ (0.05, 0.5, and 1 mM) and measured ROS production every 30 minutes for 4 hours using the DCFDA/H2DCFDA-Cellular ROS assay kit. As shown in Figure 4(b), we observed a significant increased level of ROS production starting from 1 hour of treatment with the lower dose of H_2_O_2_ (0.05 mM) and continuing progressively in time-dependent manner. CAL62 cells treated with the highest concentrations of H_2_O_2_ (0.5 and 1 mM) presented a significantly increased level of ROS starting 30 minutes with the maximum peak after 1 hour and 30 minutes (Figure 4(b)).

Based on this experiment, we started using the lower dose of H_2_O_2_ (0.05 mM) to investigate the protective effect exerted by *A*PFE in our model system. Thus, we induced the oxidative stress with H_2_O_2_ (0.05 mM) treatment in CAL62 cells pretreated for 20 hours with *A*FPE (500 *μ*M EqC). As shown in Figure 4(c), CAL62 treated for 4 hours with H_2_O_2_ presented a high production of ROS, while CAL62 pretreated with *A*FPE presented a reduction in the generation of ROS compared to cells treated with H_2_O_2_ alone. Additionally, we also found that the pretreatment with *A*FPE protects cells from the ROS production induced by highest doses of H_2_O_2_ (1 mM) (Figure 4(c)).

Overall, these results provided evidence that *A*FPE exerts antioxidant activity by inhibiting ROS production in thyroid cancer cell lines.

### 3.7. Characterization of the Protective Effect of *A*FPE on Oxidative Stress

Next, we also further characterize the protective role of *A*FPE in oxidative stress in thyroid cancer cell line by evaluating early, late apoptosis or necrosis. To this aim, CAL62 cells were seeded in medium containing 10% FBS in 6-well plates at the density of 1 × 10^5^ cells/well and the day after treated with *A*FPE (500 *μ*M EqC) for 24 hours alone or pretreated with *A*FPE (500 *μ*M EqC) for 20 hours and then treated with H_2_O_2_ (1 mM) for 4 hours or only treated with H_2_O_2_ (1 mM) for 4 hours. The percentage of cell death was examined by flow cytometry analysis with 7-AAD staining.

**Figure 5:**
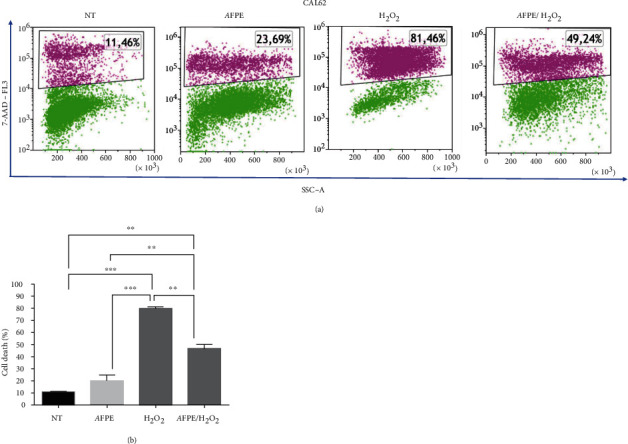
Effects of *A*FPE treatment against H_2_O_2_-induced oxidative stress in anaplastic thyroid cell line. CAL62 cell line was pretreated with *A*FPE, and then, the oxidative stress was induced by H_2_O_2_ treatment. After 24 hours, the percentage of dead cells (%) was determined by flow cytometry. (a) Representative fluorescence-activated cell sorting (FACS) plots are shown from one of two independent experiments. (b) Bar plot of the experimental data, values are expressed as mean of two different experiments ± standard error of the mean (SEM). ^∗∗^*p* < 0.05; ^∗∗∗^*p* < 0.001. NT: untreated cells; H_2_O_2_: cells treated with hydrogen peroxide; *A*FPE: cells treated with *A*FPE alone; *A*FPE/H_2_O_2_: cells pretreated with *A*FPE and stressed using hydrogen peroxide. For the multiple comparisons, statistical analysis between the four experimental groups (NT, *A*FPE, H_2_O_2_, and *A*FPE/H_2_O_2_) and ANOVA followed by a post hoc test (Bonferroni) were performed.

As shown in Figure 5, the treatment with *A*FPE (500 *μ*M EqC) alone induced an increase in cell death percentage that raised from 11.3% to 20.2% in CAL62 cells; H_2_O_2_ alone caused a significant increase in the percentage of cell death resulting to be approximately 79% in CAL62, while in the cells pretreated with *A*FPE, cell death percentage was approximately 46%.

**Figure 6:**
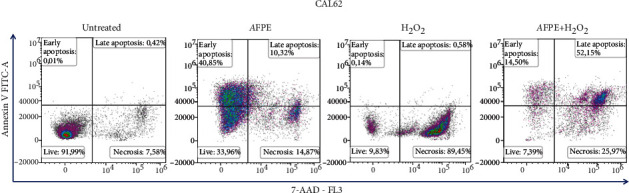
Effects of *A*FPE on apoptosis and necrosis in CAL62 cells. CAL62 cells were treated with *A*FPE (500 *μ*M EqC), H_2_O_2_ (1 mM), or the combination as described above. Early and late apoptosis and necrosis were evaluated by staining cells with Annexin V and 7-AAD and by analyzing with flow cytometry. Shown are early (Annexin V positive/7-AAD negative) and late (Annexin V positive/7-AAD positive) apoptotic and necrotic (Annexin V negative/7-AAD positive) cells.

Then, to better characterize the effects on cell death, we stained CAL62 cells treated with AFPE (500 *μ*M EqC) or H_2_O_2_ (1 mM) or the combination with both Annexin V and 7-AAD and analyzed the results by flow cytometry. CAL62 cells treated with only *A*FPE disclosed: 40.85% of early apoptosis (Annexin V positive/7-AAD negative), 10.32% of late apoptosis (Annexin V positive/7-AAD positive), and 14.87% of necrosis (Annexin V negative/7-AAD positive). Treatment with H_2_O_2_ induced mainly cellular necrosis (89.45%) and not apoptosis. *A*FPE pretreatment could counteract H_2_O_2_ oxidative damage; indeed, we found that cells treated with *A*FPE for 20 hours and then with H_2_O_2_ for 4 hours show 14.5% and 52.15% of, respectively, early and late apoptosis. Furthermore, *A*FPE pretreatment reduced the percentage of necrotic cells to 25.97% in comparison to 89.45% detected in H_2_O_2_ treated cells (Figure 6).

**Figure 7:**
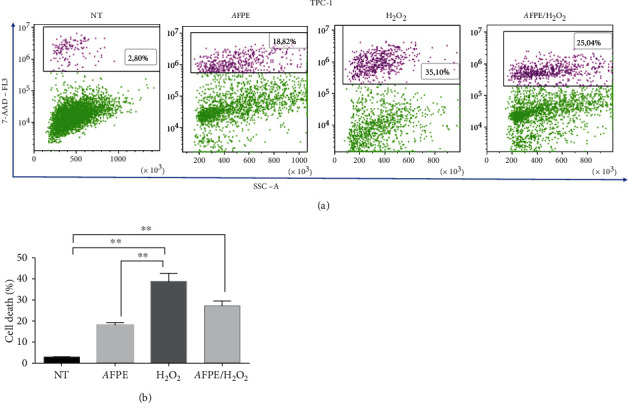
Effects of *A*FPE treatment against H_2_O_2_-induced oxidative stress in papillary thyroid cell line. TPC-1 cell line was pretreated with *A*FPE, and then, the oxidative stress was induced by H_2_O_2_ treatment. After 24 hours, dead cells (%) were determined by flow cytometry. (a) Representative plots of cell death analysis obtained by fluorescence-activated cell sorting (FACS). One of two independent experiments is shown. (b) Bar plot of the experimental data, values of percentage of dead cells are expressed as mean of two different experiments ± standard error of the mean (SEM). ^∗∗^*p* < 0.01. NT: untreated cells; H_2_O_2_: cells treated with hydrogen peroxide; *A*FPE: cells treated with *A*FPE alone; *A*FPE/H_2_O_2_: cells pretreated with *A*FPE and stressed using hydrogen peroxide. For the multiple comparisons, statistical analysis between the four experimental groups (NT, *A*FPE, H_2_O_2_, and *A*FPE/H_2_O_2_) and ANOVA followed by a post hoc test (Bonferroni) were performed.

Then, we analyzed the protective effect of AFPE on oxidative stress in TPC-1 cells. As shown in Figure 7, the treatment of TPC-1 cells with *A*FPE (500 *μ*M EqC) alone induced an increase in cell death that raised from 2.9% to 17.9%. H_2_O_2_ alone caused a significant increase in the percentage of cell death resulting to be approximately 38%, while in the TPC-1 cells pretreated with *A*FPE, cell death percentage was approximately 27% (Figure 7).

These results provided evidence that, in human thyroid cancer cells, death induced by oxidative stress was reduced by the pretreatment with *A*FPE.

## 4. Discussion

Phytochemicals are compounds available in plant-including foods characterized by antioxidant and antitumor abilities [6, 18]. Among these natural substances, polyphenols in apple fruit could have an important role in preventing cancer by exerting antioxidant activity [7, 29]. Indeed, the beneficial effects of these compounds are attributable, at least in part, to their ability to scavenge the ROS [44, 50, 51]. However, in other context, polyphenols act also as prooxidant agents since they are able to generate ROS and induce cellular oxidative stress and, consequently, cell death [39, 41, 52, 53].

In addition to these abilities, several *in vitro* studies showed that in human cancer cell lines, apple polyphenols inhibit cell proliferation and invasion [35, 43, 54].

For all these reasons, the growing scientific interest is focused on identifying the biological mechanisms and the signal transduction pathways underpinning the chemo-preventive activities of these compounds.

Pathological conditions of the thyroid are often associated with oxidative stress that results mainly from excessive production of ROS, including H_2_O_2_ possibly concurring to cell transformation and genomic instability [55]. Since the thyroid follicular cells require H_2_O_2_ to synthesize thyroid hormones (T_3_ and T_4_), in thyroid gland, it is very difficult to regulate the balance between ROS production and scavenging [55]. Indeed, despite the rate of ROS production is efficiently controlled and removed by natural repair mechanisms, it can occur that the endogenous antioxidant systems are not sufficed. These mechanisms may be supported by exogenous substances with genoprotective and antioxidative activity that can be provided to the organism from a diet rich in food of plant origin [56, 57]. Interestingly, it was observed that polyphenols such as genistein, resveratrol, and quercetin might be effective for thyroid cancer therapy, including for anaplastic thyroid cancer [14, 26, 58].

In this context, within this study, we aimed to deepen the knowledge on the effects of polyphenol extracts obtained from *Annurca* apple flesh (*A*FPE). We have observed that when *A*FPE was administered for 24 hours, the proliferation of thyroid cancer cell lines (CAL62 and TPC-1) was inhibited in a dose-dependent manner. By flow cytometry, we also found that *A*FPE treatment induced cell death in both CAL62 and TPC-1 cell lines. Coherently, with our results, in breast cancer, *A*FPE inhibits cell growth, causes cell cycle arrest, and induces apoptosis in MDA-MB-231 cell line [41].

Here, we also investigated the molecular mechanisms underlying the effects observed following *A*FPE treatments in CAL62 cell line, and we found that the anticancer activity induced by *A*FPE exposure is associated with deregulated expression of several miRNAs which are known to be involved in thyroid cancer and in mediating the effects of polyphenol. Indeed, we found that the expression level of miR-141, miR-145, miR-200a-5p, miR-425, and miR-551b-5p resulted upregulated in CAL62 treated with *A*FPE with respect to untreated cells.

In literature, several papers reported that miR-145 is downregulated in thyroid cancer where it acts as a tumor suppressor by impairing cancer cell growth, cell motility, and metastasis through targeting AKT3 [59] and RAB5C [60] genes or by regulating the NF-*κ*B pathway [61].

Interestingly, in our cell model system, we found that miR-141 and miR-200a-5p are increased after *A*FPE exposure. It is well known that the members of miR-200 family (miR-200a, miR-200b, miR-200c, miR-141, and miR-429) play an important role in thyroid cancer. In thyroid cancer, it is reported that the loss of miR-141 and of miR-200a expression is associated with thyroid cancer proliferation, invasion, and metastasis [62–64]. It is also reported that the restoration of miR-141 and of miR-200a expression induced an epithelial morphology in ATC cells [65].

Concerning miR-425, upregulated by *A*FPE in CAL62 cells, in literature is described to be decreased in PTC tissues; furthermore, the transfection of miR-425 mimic decreases the motility phenotype of PTC cells [66]. Interestingly, the aberrant expression of miR-425 is also correlated to the oxidative stress in breast cancer cell lines where it regulates the apoptosis by PTEN axis [67, 68]. Indeed, it has been recently highlighted that miRNA plays an important role in the homeostasis of ROS during carcinogenesis [67, 69, 70].

Finally, previous studies revealed that the polyphenols exert their health benefits in mouse model by regulating the expression of several miRNAs including miR-551b-5p that we also found to be upregulated in CAL62 following the *A*FPE exposure [71, 72].

Concerning other molecular mechanisms deregulated by *A*FPE in thyroid cancer cells, there are evidences that *A*FPE inhibits the expression and activity of several oncoproteins related to cell survival and proliferation such as AKT, NF-*κ*B, and *β*-CATENIN [41]. These oncogenic pathways are also altered in thyroid cancer, suggesting a possible negative regulation of them by *A*FPE exposure [73–76].

Here, we have also investigated the effects of *A*FPE treatment against H_2_O_2_-induced oxidative stress in papillary and anaplastic thyroid cell lines. Our *in vitro* results show that, in human thyroid cancer cell lines, cell death induced by oxidative stress was reduced by the pretreatment with *A*FPE.

The knowledge regarding the polyphenols as modulator of oxidative stress based on their differential redox status is already documented in human cancer [77].

This could offer a window of opportunities in thyroid cancer since *A*FPE can lead to apoptosis and cell cycle arrest of malignant cells, but it is also able to protect human cells from the DNA breakage induced by reactive oxygen species.

Thus, based on these findings, this study suggest that development of phytochemical could be important for thyroid cancer treatment.

A limitation of this work is the high concentration of *A*FPE used in the thyroid cancer cell lines that are above those achievable *in vivo*. It should be also pointed out that all the experiments described here have been performed in medium containing 10% serum.

Polyphenols are low bioavailability compounds with poor solubility in water. The absorption in the colon is different for the diverse groups of polyphenols; indeed, the flavan-3-ol derivates demonstrate higher absorption rate compared to quercetin [78]. Polyphenols are absorbed by the gut as aglycones and then released into the blood after chemical change that modifies their effects [79]. During gastrointestinal digestion, several modifications occur that could affect their bioavailability, stability, and bioactivity [80].

Importantly, the bioavailability of polyphenols is limited by their extensive metabolism [81, 82]. Additionally, several factors influence the bioavailability of polyphenols: environmental factors, food processing, interaction with other compounds, genetic factors, the composition of microflora, and the length of the microbiome [83, 84]. Indeed, Wruss et al. studied the pharmacokinetic of polyphenols in apple juice, in healthy subjects, and discovered numerous differences in apple polyphenol pharmacokinetics [85]. Also, Tenore and colleagues evaluated the bioaccessibility and bioavailability of *A*FPE, by simulating an *in vitro* gastrointestinal digestion [86]. Moreover, novel biotechnologies for improving bioavailability of polyphenols have been developed based on different approaches. For example, the encapsulation of polyphenols could contribute to the intensification in their shelf life and could avoid their loss in activity, thus improving the bioavailability [87]. Notwithstanding, further studies are necessary on the bioavailability and bioaccessibility of polyphenols with the aim of developing novel strategies for the production of functional foods.

## 5. Conclusion

In conclusion, our findings suggest that *A*FPE could be a promising tool for thyroid cancer treatment. Indeed, we found that *A*FPE acts on cell proliferation and viability. On the other hand, *A*FPE also acts as an antioxidant modulator protecting cells from oxidative stress in the presence of oxidant agent such as peroxide hydrogen. Here, we have also clarified some of the biological mechanisms involved in the effects of this polyphenol in thyroid cancer, as deregulation of several cancer-related miRNAs and decreased phosphorylation of Rb protein. Nevertheless, other studies are needed to better dissect the molecular mechanisms underlying the effects of *A*FPE on the thyroid cancer cells.

## Data Availability

The data underlying this study are included in the article; further inquiries can be directed to the corresponding authors.

## Abbreviations

7-AAD: 7-Amino-actinomycin D (7-AAD)
ATC: Anaplastic thyroid cancer
*A*FPE: *Annurca* flesh polyphenolic extract
CatEq: Catechin equivalent
DMEM: Dulbecco's modified Eagle's medium
FACS: Fluorescence-activated cell sorting
FBS: Fetal bovine serum
PI: Propidium iodide
PBS: Phosphate-buffered saline
PTC: Papillary thyroid cancer.
